# Mapping the yearly extent of surface coal mining in Central Appalachia using Landsat and Google Earth Engine

**DOI:** 10.1371/journal.pone.0197758

**Published:** 2018-07-25

**Authors:** Andrew A. Pericak, Christian J. Thomas, David A. Kroodsma, Matthew F. Wasson, Matthew R. V. Ross, Nicholas E. Clinton, David J. Campagna, Yolandita Franklin, Emily S. Bernhardt, John F. Amos

**Affiliations:** 1 Department of Biology, Duke University, Durham, North Carolina, United States of America; 2 SkyTruth, Shepherdstown, West Virginia, United States of America; 3 Appalachian Voices, Boone, North Carolina, United States of America; 4 Google Earth Engine Team, Google Inc., Mountain View, California, United States of America; 5 Department of Geology & Geography, West Virginia University, Morgantown, West Virginia, United States of America; Universidade de Vigo, SPAIN

## Abstract

Surface mining for coal has taken place in the Central Appalachian region of the United States for well over a century, with a notable increase since the 1970s. Researchers have quantified the ecosystem and health impacts stemming from mining, relying in part on a geospatial dataset defining surface mining’s extent at a decadal interval. This dataset, however, does not deliver the temporal resolution necessary to support research that could establish causal links between mining activity and environmental or public health and safety outcomes, nor has it been updated since 2005. Here we use Google Earth Engine and Landsat imagery to map the yearly extent of surface coal mining in Central Appalachia from 1985 through 2015, making our processing models and output data publicly available. We find that 2,900 km^2^ of land has been newly mined over this 31-year period. Adding this more-recent mining to surface mines constructed prior to 1985, we calculate a cumulative mining footprint of 5,900 km^2^. Over the study period, correlating active mine area with historical surface mine coal production shows that each metric ton of coal is associated with 12 m^2^ of actively mined land. Our automated, open-source model can be regularly updated as new surface mining occurs in the region and can be refined to capture mining reclamation activity into the future. We freely and openly offer the data for use in a range of environmental, health, and economic studies; moreover, we demonstrate the capability of using tools like Earth Engine to analyze years of remotely sensed imagery over spatially large areas to quantify land use change.

## Introduction

### Impacts of surface coal mining in Appalachia

Surface mining is a broadly used mining technique that has increasingly replaced underground mining for a variety of resources [1], especially coal in the United States [2]. In Central Appalachia, most of this surface mining for coal is done in the steep, dissected landscapes of Kentucky, Tennessee, Virginia, and West Virginia. Surface mining in such steep landscapes is called mountaintop removal coal mining with valley fills (MTMVF). To access coal from the surface, MTMVF operators harvest overlying forest, dismantle bedrock with explosives and heavy machinery, and extract coal seams ranging from 0.25 through 1.5 m thick [3]. This process generates large quantities of leftover waste rock, or mine spoils, which are deposited into headwater valleys, burying streams in as much as ~200 m of spoil [4]. Unlike many other types of surface mine operations, which may be hundreds of meters deep but occur over relatively small spatial scales, MTMVF mines have been constructed across thousands of square kilometers of land, making it the single largest source of land use change in the region [5,6].

MTMVF dramatically alters vegetation, surface topography, and subsurface structure in mined regions. Native Appalachian forests do not reestablish on most post-mining landscapes, causing a shift from forest to grassland/shrubland ecosystems [7,8]. These non-native ecosystems grow on a landscape where mining has lowered the local topographic complexity [9], lowered the average slope by nearly 10° [4], and created novel plateau-like landscapes [4,9]. Changing Appalachian landscapes from steep, shallow-soiled forests to flat grasslands overlying deep spoil piles has altered how water and elements move through these landscapes [10].

In streams draining valley fills, the flat landscapes and increased storage potential have been shown to lower stream discharge during storm events and elevate baseflow [11]. The water stored in these valley fills is steeped in a reactive matrix of pyrite and carbonaceous bedrock. Pyrite, bound up in coal residue and shales, produces sulfuric acid when exposed to oxygen and water [12]. In MTMVF spoils, this sulfuric acid is neutralized by carbonate materials, which are intentionally mixed with spoils to prevent acid-mine drainage [13,14]. The ready supply of sulfuric acid, carbonate bedrock, and high surface-area spoil materials creates ideal conditions for some of the highest weathering rates in the world. The net weathering reactions generate alkaline mine drainage which is characterized by elevated ion concentrations of sulfate (SO_4_^2-^), calcium (Ca^2+^), magnesium (Mg^2+^), bicarbonate (HCO_3_^-^), and a suite of other elements including major aquatic pollutants like selenium (Se) [15,16]. The increased ion concentrations raise the mean specific conductance of water in mined streams from background values below ~200 to averages well over 1,000 μS/cm [15].

Throughout Central Appalachian streams, the physicochemical impacts from mining operations have been shown to decrease aquatic macroinvertebrate diversity [17,18,19], alter microbial communities [20], negatively impact fish [15,16], lower salamander abundance [21], and decrease stream leaf-litter breakdown rates [22]. In addition to these negative aquatic impacts, MTMVF has been shown to substantially increase the carbon cost of burning coal [23], fragment forest habitat [24,25], and elevate local surface temperature [24]. Finally, mining operations can mobilize significant dust clouds with particulates that can cause detrimental human health impacts [26].

### Mapping mining extent

Despite the overwhelming and widespread negative consequences from MTMVF, the exact extent of mining operations in the Appalachian region has not been updated since 2005 [27]. That dataset (hereafter referred to as the “MTM2009” dataset after its year of publication) identifies surface mines at a 30 m resolution, detectable as early as 1976 and as late as 2005, across most of Central Appalachia. The MTM2009 dataset’s major limitation, however, is its temporal resolution; it only maps mining operations at ~ decadal timescale. While the dataset specifies exactly where a mine has occurred, it provides a coarse ten-year window of when that mine may have been in active production. To parse the hydrologic, biogeochemical, ecological, and human-health impacts from mining, researchers require finer temporal resolution maps of mining extent.

Here, we aim to improve upon the ten-year MTM2009 dataset by offering a yearly, 30 m dataset covering the period 1985 through 2015, and to make these data freely and publicly accessible for any use. In particular, we are interested in locating areas being actively mined in any given year. We broadly define active mining as any land where mining activity (i.e., earth removal and replacement) was likely occurring, or where mining activities had recently ceased so that the landscape still resembled a mine in active development.

Moreover, we aim to automate the modeling process so that future mining areas can be quickly added to the dataset as new remotely sensed imagery becomes available. The MTM2009 dataset relied on a time-intensive supervised classification, but for our updated approach we sought to develop automated methods that would enable annual estimates of past mining and facilitate rapid updating of the dataset in the future. Such automation is facilitated in this case because the spectral characteristics of a surface mine vary considerably from other land cover classes in this region.

To model mining extent, we use the Google Earth Engine platform. Earth Engine is a freely accessible, cloud-based Google product designed to enable remote sensing studies over long time scales and large spatial extents. In addition to running data processing operations, Earth Engine hosts full collections of public remote sensing data. In our case, we use Earth Engine’s processing capabilities and its continually updated archive of Landsat imagery to produce our dataset.

While Earth Engine is itself relatively new, many researchers have used moderate resolution Landsat imagery to quantify land cover changes caused by surface mining in Appalachia and elsewhere. For instance, researchers acquired one Landsat image per year for a four-county area in Virginia to identify mine lands at a yearly time scale from 1984 through 2001 [10]. Other researchers used Landsat imagery for four years at a decadal interval to identify surface mines and reclamation activity [5]. A different study compared Landsat imagery and land cover data from 1992 and 2001 to determine that surface mining had caused an accelerated loss of ecologically important interior forest in Appalachia [24]. Yet another used Landsat imagery to show that species planted to aid with mine reclamation, such as the invasive autumn olive, could be identified over previous surface mines via remote sensing [28]. And finally, the LandTrendr product, a set of algorithms to quantify pixel-level land cover change over time, employs Landsat imagery for its analysis [29].

Here we use Earth Engine to create an automated model that identifies surface coal mining, particularly MTMVF, across Central Appalachia (a contiguous area including portions of Kentucky, Tennessee, Virginia, and West Virginia) at a yearly time scale. We call our approach “automated” because the algorithms only require the user to supply the raw, orthorectified Landsat scenes; the user does not need to manipulate additional parameters. We then present summary statistics from the model, explore the model’s accuracy, and compare the model to findings from the MTM2009 dataset. We demonstrate one example of using these data in combination with other datasets, in this case to correlate mined area with coal production. We explain some dataset limitations and conclude with suggestions for future enhancements to our model.

## Materials and methods

### Study area

**Fig 1:**
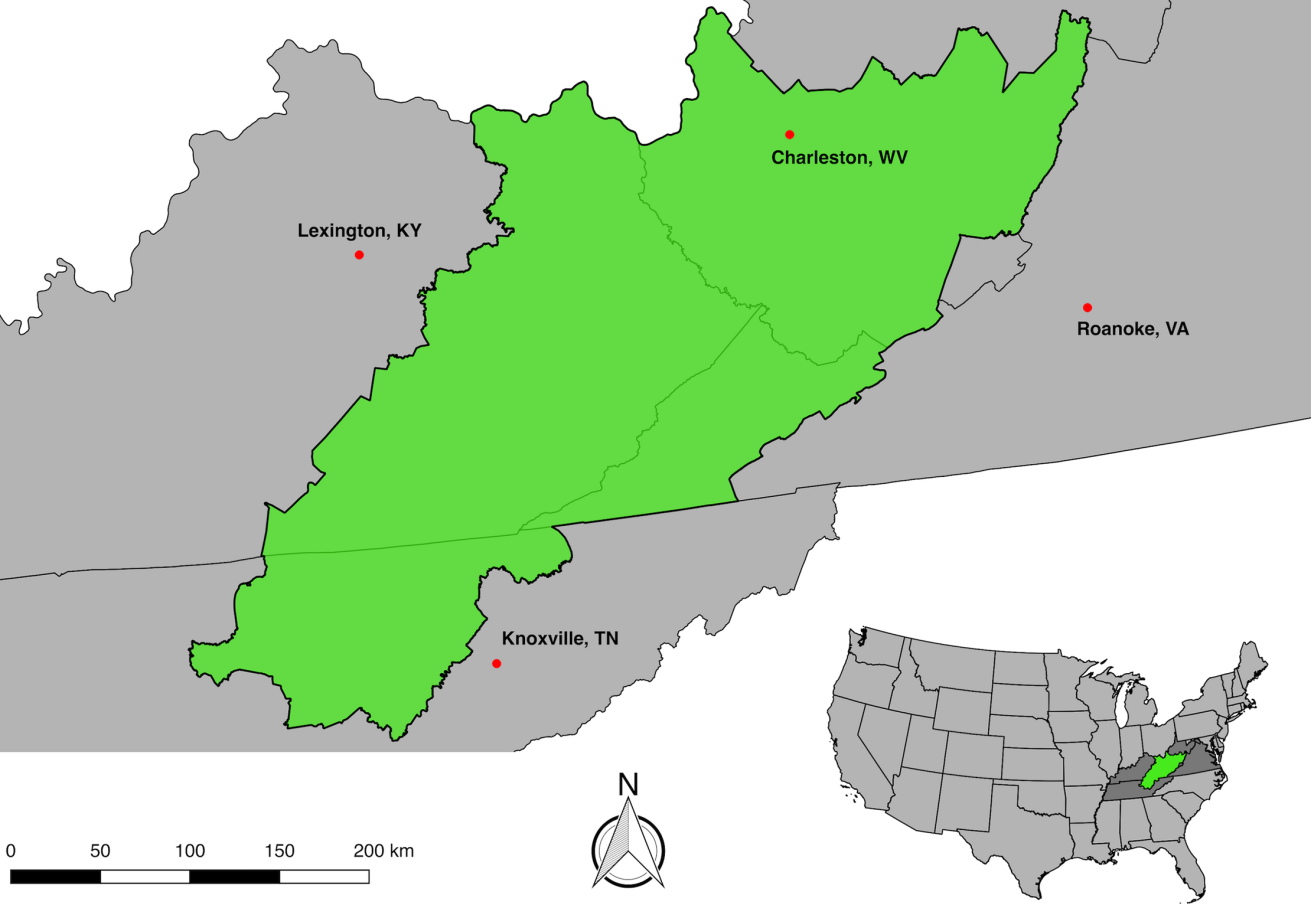
Map of study area. The study area ranges in latitude from 35.6444° to 39.0298° and in longitude from -79.6179° to -85.8093°. Geospatial data describing the study area and the ultimate model outputs are available to download at https://www.skytruth.org/mtr-data-files/.

We chose a 74-county, 83,000 km^2^ area in Central Appalachia, composed of counties in Kentucky, Tennessee, Virginia, and West Virginia, to conduct our model (Fig 1). Our model ultimately processed imagery covering the entirety of this study area. Surface coal mine production has been reported to the United States Mine Safety and Health Administration in all of these counties at some point since 1983 [30], and all of these counties are within the Central Appalachian Basin as defined by United States Geological Survey [31].

### Analysis model

We carried out Landsat data processing by writing JavaScript processing scripts using the Google Earth Engine’s application programing interface (S1 File); these scripts cleaned each input scene (i.e., image) for data abnormalities or cloud cover; determined the normalized difference vegetation index (NDVI) per scene; derived a greenest-pixel (maximum NDVI) composite per year; and labeled each pixel within each composite as likely active mine or likely non-mine based on an annual, county-scale NDVI threshold that was computed as described below. We more narrowly define active mine areas as those locations with a model-indicated low NDVI as compared to the NDVI of nearby, forested areas. This simple classification relies on the stark spectral contrast in this Appalachian region between mines and forests: our model identifies areas largely devoid of vegetation and calls them mines. Our output dataset is thus a series of annual, 30 m pixel resolution, binary images depicting locations where mining likely occurred throughout the given year.

**Fig 2:**
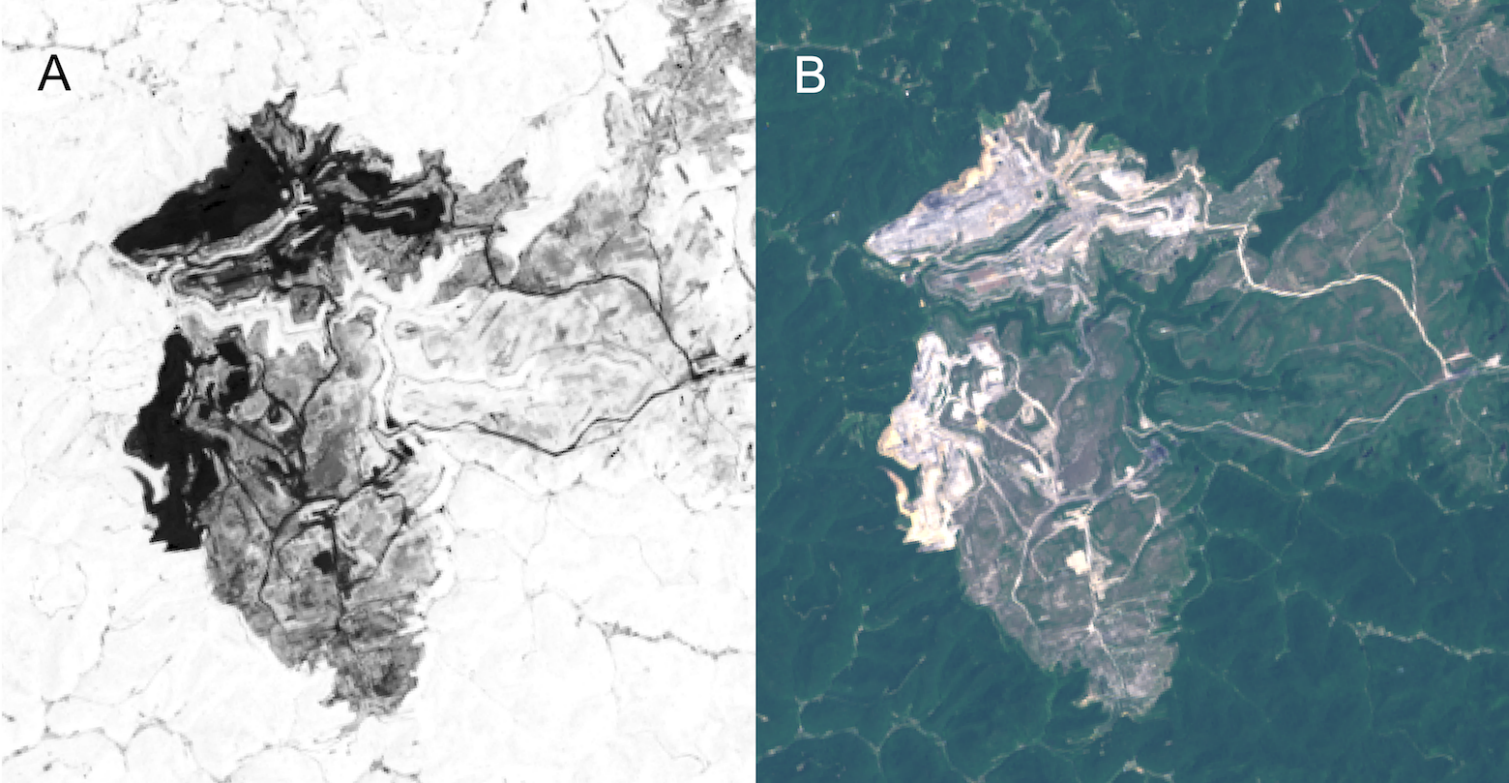
Example NDVI image and associated true color image. These images from May 2014 show the area near Spurlockville, WV, and in particular the Hobet-21 mountaintop coal mine (38.08°, -81.95°). Darker colors in the NDVI image (A) indicate lower NDVI values. True color imagery (B) demonstrates that, in the visual spectrum, forested areas appear green whereas mined areas appear gray. Both images are derived from Landsat 8 top-of-atmosphere reflectance imagery and were processed in Earth Engine for visualization.

NDVI images depict areas with high levels of photosynthetically active vegetation. Calculating NDVI is simple, requiring only a red and near-infrared reflectance value per pixel [32]. A vegetated pixel will have a high NDVI value (near 1.0), whereas a non-vegetated pixel will have a low NDVI (near -1.0). We expect to see high NDVI values across the Appalachian landscape, especially in relatively undisturbed areas. Conversely, highly disturbed areas such as surface mines will have a relatively low NDVI compared against a vegetated background (Fig 2).

We cleaned a collection of raw Landsat scenes for undesirable pixels like clouds or sensor errors; to exclude areas of non-vegetated disturbance unlikely to be mines, such as urban areas or roads, we compiled spatial information from publicly accessible datasets and masked out those pixels. From those cleaned and masked images, we determined each pixel’s maximum NDVI per year to form a series of annual “greenest-pixel” images. These images show the greatest photosynthetic activity at any given pixel over the course of that year. The number of cleaned Landsat scenes used to create these greenest-pixel composites varied by pixel and by year, ranging from as few as one image per pixel per year to more than 30, owing to factors like low image frequency or frequent cloud cover. By choosing the maximum NDVI value in creating the composites, mines that were established over the course of that year would likely not appear in the greenest-pixel image; a green, forested pixel from earlier that year would likely have a higher NDVI value than the mine pixel, and consequently that forested pixel would appear in the composite.

For each masked greenest-pixel composite image, we established county-level NDVI thresholds that allowed us to sort remaining pixels into likely mines versus other types of surface covers. The processing scripts determined the thresholds by collecting the NDVI values of pixels per county not within a known mine permit boundary (i.e., likely forested pixels) and setting the threshold at the 0^th^ to 3^rd^ percentile mean of those pixels. In other words, we identified nearly the lowest NDVI of known forested areas and assumed any pixels with NDVI values less than that minimum value were likely mines. We used a county scale to reflect spatial differences in image quality, as well as natural landscape variation over space.

We then cleaned the resulting binary images to remove any null values or to remove very small areas labeled as mines. In particular, if our model identified a pixel at year *n* as a mine but identified that same pixel at both years (*n*-1) and (*n*+1) as other surface cover, we reclassified the pixel at year *n* as non-mine. Likewise, we reclassified any non-mine pixel at year *n* as a mine if that pixel was identified as a mine in years (*n-*1) and (*n*+1). Finally, we removed mine patches less than 9,000 m^2^.

We assessed the accuracy of our dataset by comparing manually classified points to the classifications determined by our model. Per year, we gathered true- and false-color imagery from Landsat or from the United States Department of Agriculture’s National Agriculture Imagery Program, when available. We established 10, 250 km^2^, circular plots randomly throughout the study area, ensuring that each study plot contained some active mining. We then randomly distributed and visually classified a minimum of 2,000 points per year and took a subset of that classification so that each plot in that year contained at least 150 non-mine points and 50 mine points, or a total of approximately 62,000 classified locations over 31 years. We used these data to assess the model accuracy on an annual basis.

### Incorporating MTM2009 data

Since our model started at 1985 but we know MTMVF has occurred prior to that date, we incorporated the 1976 through 1984 subset of the MTM2009 data into our dataset. We selected only those pixels identified as mines in 1976 or 1984 from the MTM2009 data and spatially appended them to our 1985 through 2015 cumulative mining dataset. We thus generated a “first-mined” dataset that reveals whether a certain area was first converted into a surface mine either by 1976, between 1977 and 1984, or in any year from 1985 through 2015. Of note, many mines labeled by MTM2009 as “1976” likely started at some unknown date prior to 1976, so we cannot precisely say when those earliest mines began. We likewise generated a “last-mined” dataset that says when a given area was most recently an active mine; however, this dataset cannot show if a given area was once mined but became reclaimed (i.e., ceased mining) and later became a mine again. Our results below are based off the “first-mined” dataset or the annual mining dataset generated in this study alone.

## Results and discussion

### Total mining extent

**Fig 3:**
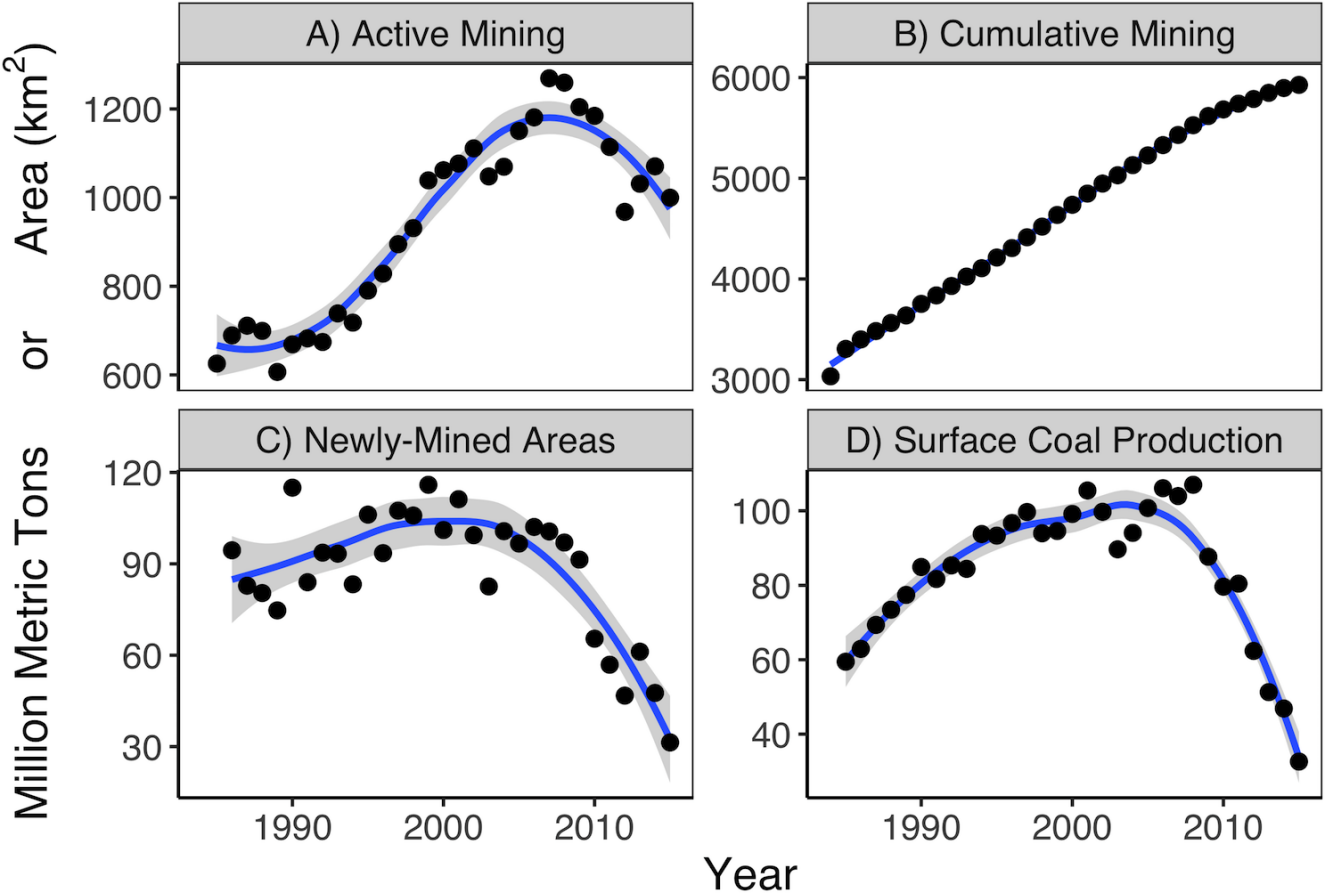
Active mining, cumulative mining, and coal production over time. “Active Mining” (A) means any land area detected by our model as likely mine for the given year; “Cumulative Mining” (B) is the non-duplicative summation of active mine area over time; this sum includes mine areas identified from pre-1976 through 1984 from the MTM2009 data (see above). “Newly-Mined Areas” (C) is the land area that was first converted into a mine in the given year. “Surface Coal Production” (D) data is from the Mine Safety and Health Administration [30] rather than our model; we present it here for comparison.

Between 1985 and 2015, an average of 87 km^2^ of previously unmined land was converted to a surface mine in any given year, with this annual rate of change varying from a low of 31 km^2^ yr^-1^ in 2015 to 116 km^2^ yr^-1^ in 1999 (Fig 3C). Over time, this adds up to a total of approximately 2,900 km^2^ (or approximately 3.5 percent) of Central Appalachia that has been part of an active surface coal mine at some point between 1985 through 2015 (Fig 3B). Rates of both new mine area expansion and coal production (Fig 3C & 3D) have dropped off precipitously since 2010.

The full scope of cumulative mining from 1976 through 2015, incorporating the 1976 and 1984 subset of the MTM2009 data as discussed above, yields a total of 5,900 km^2^ of detectable mining over the 40-year period (Fig 3B). This total indicates that 3,000 km^2^ was first mined in either pre-1976 or 1984. For comparison, the cumulative surface mining area from pre-1976 through 2015 comprises 7.1 percent of Central Appalachia, and is 18 percent larger than the land area of the state of Delaware and only 3.3 percent smaller than the area of Everglades National Park.

We demonstrate that cumulative mining increases at a near-linear rate from 1985 through 2015, increasing on average 87 km^2^ yr^-1^ (a new mine “conversion rate” from previously unmined to mined land). However, between 1984 (i.e., the end of the MTM2009 data) and 1985, this cumulative total jumps more than 270 km^2^ (Fig 3B). In other words, our results suggest 270 km^2^ of land previously unmined through 1984 suddenly became a mine in 1985, but then that rate dropped to an average of 87 km^2^ yr^-1^ after 1985.

This seeming discrepancy is likely a result of combining our mining detection algorithm with the MTM2009 data, and not a single year dramatic increase in mining rates. On one hand, our study area is larger than that of the MTM2009 study. While many of the largest mines were identified by both studies, our somewhat larger study area could have given the appearance of much new mine area in 1985 by finding mines simply not located within MTM2009’s study area. Additionally, we acknowledge that our automated model is likely more lenient in deciding if a pixel represents a mine than was the supervised classification approach employed by the MTM2009 study. For example, pixels on the edge of a large mine area may have been labeled as mines by our study but non-mines by MTM2009, leading to a further increase in our areal total by 1985.

### Annual active mining extent

Whereas the MTM2009 study only shows where mining occurred during some ten-year period, our study reveals the yearly areas that were actively being mined. Over the period 1985 through 2015, we find an average of 940 km^2^ (greater than 1.1 percent) of the study area under active mining in any given year (Fig 3A). Active mining ranges from 610 km^2^ to 1,300 km^2^ per year. The change in active mining area per year is highly irregular, ranging from an additional 110 km^2^ of mining between one year and the next to a decrease of 150 km^2^.

**Fig 4:**
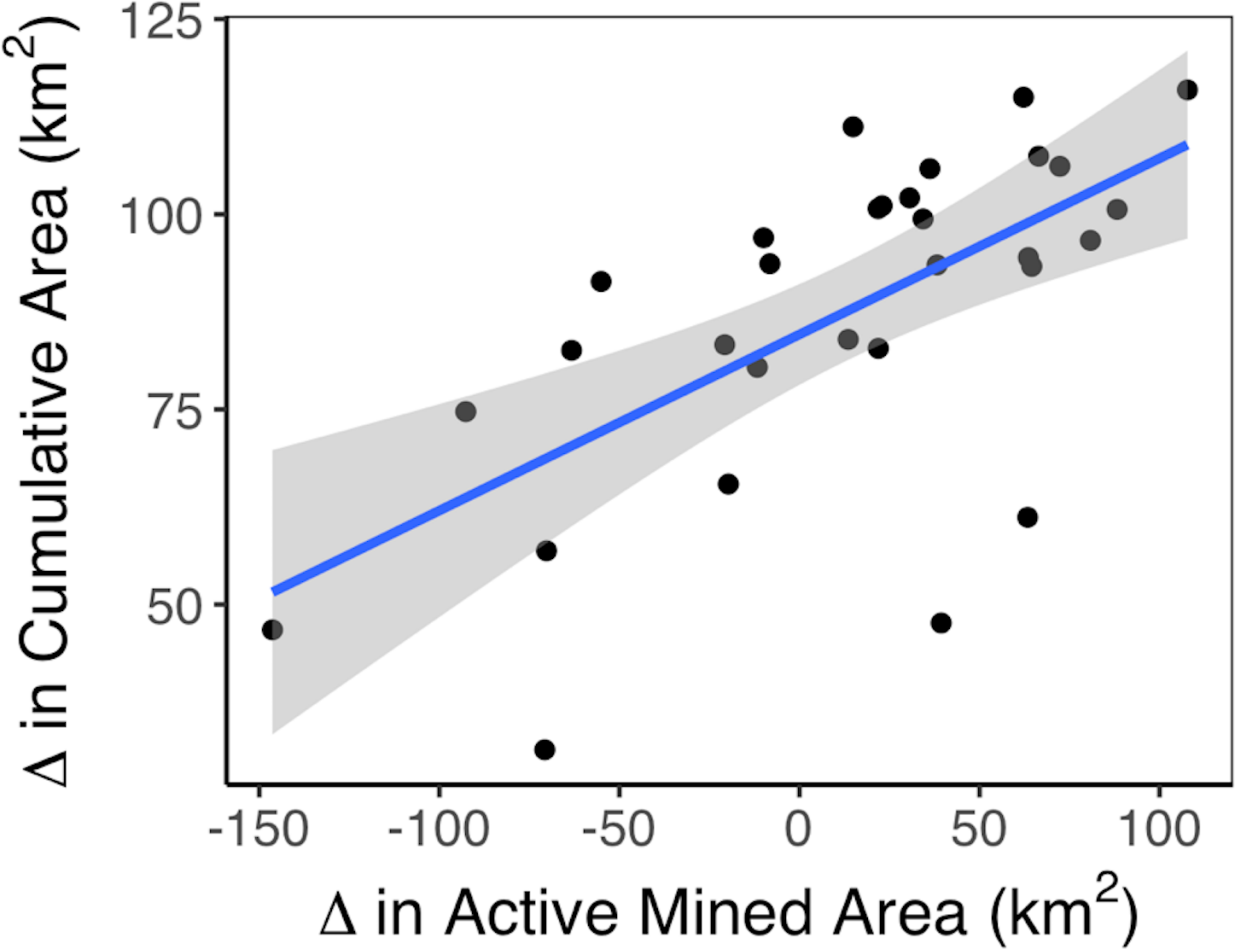
Annual change in cumulative mining versus change in active mining. “Active Mined Area” is any area per year where the maximum NDVI observed in that year was less than the NDVI threshold set per county per year. “Cumulative Area” is the summation of unique active mine area over time; if some location was identified as active mine in one year, its area would not be added again to the cumulative mining total in future years. Each 1 m^2^ of land in active mining is associated with 0.22 m^2^ of newly mined area.

To explain this irregular change, however, we find a moderately strong, positive relationship (*r* = 0.63) between the change in active mining in any given year and the change in cumulative mining in that year (Fig 4). This relationship suggests that years with much active mining also had much newly mined land (i.e., a large increase in the cumulative area); and that years with little active mining had little newly mined land (i.e., a small increase in the cumulative area). In this region of Appalachia, when mine companies put forth heightened mining effort, that effort went in general toward mining new lands rather than re-mining old lands. Moreover, we regressed these data to show that every 1 m^2^ of land under active mining is significantly associated with 0.22 m^2^ of conversion to newly mined area (*p* < 0.001, *r*^2^ = 0.40). In other words, approximately one-fifth of active mine land in any given year represents newly converted area.

### Accuracy assessment

We find that our NDVI-based model accurately and efficiently reveals yearly mining extent (S1 Table). Our accuracy assessment yielded values for the Cohen’s Kappa coefficient ranging from 0.62 to 0.93 per year. These positive values suggest that, in all cases, our model performs 62 to 93 percent better than random chance. We also find that the user’s accuracy of mined points for each year is at least 0.83 or higher, indicating that at least 83 percent of pixels labeled as mines actually represent mines on the ground. Of the 31 years analyzed, 21 years have mine user accuracy values greater than or equal to 0.90.

### Comparison to mine production data

As a way to assess our model results and to explore relationships between extraction and disturbance, we investigated the degree to which our mine area totals are correlated with known coal production from surface mines in Central Appalachia. Theoretically, the land impacts of any increase or decrease in production from surface mines should show up in our mine extent data, but with a lag between production and the associated active mining area. A lag likely exists because it takes years for a cleared area to revegetate—particularly an area that has undergone the high intensity of disturbance caused by a surface coal mine. Moreover, in a large operation like a mountaintop mine where multiple coal seams may be mined successively, an area may continue to be mined for several years as mine companies blast and dig through hundreds of feet of elevation to access all of the coal seams.

We acquired coal production data provided quarterly to the Mine Safety and Health Administration by all mining companies operating in the U.S. [30]. These data report how much coal was produced by different mining techniques such as underground mining and strip mining. For this study, we only used production data reported from Central Appalachian "strip" mines, as the other techniques have very little surface impact per ton of coal produced.

**Fig 5:**
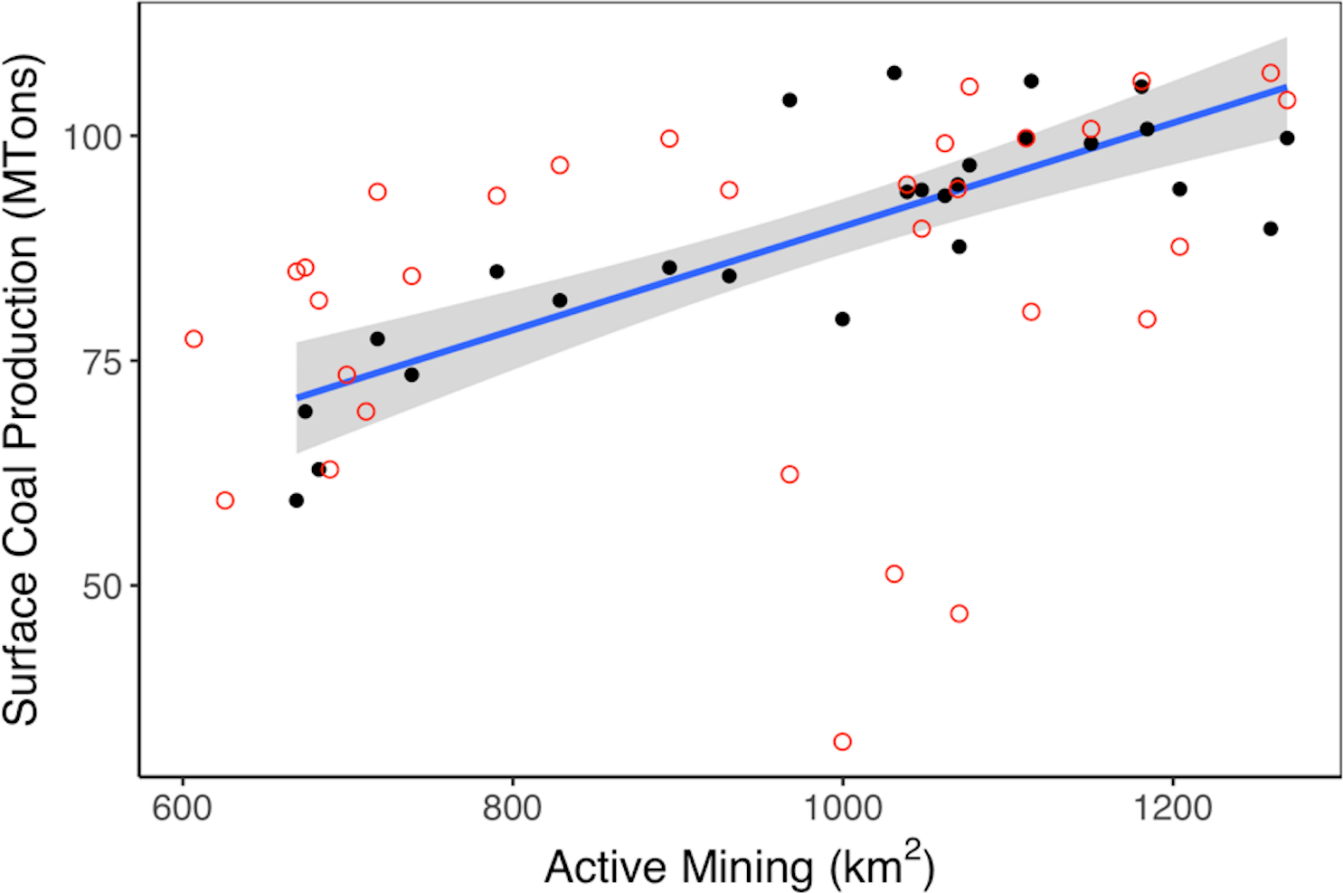
Yearly mine production versus active mine area, with and without a 5-year time lag. Rates of coal production from surface coal mines in the region are compared to the scale of active mining estimated in the same year (red circles) and five years previously (black circles). There is no relationship when data are analyzed from the same year, but the amount of coal produced five years earlier explains 68% of the variance in active mining area (regression fit shown in blue with 95% confidence interval in gray).

We regressed yearly mine production quantities with area in active mining, initially finding virtually no model fit when assuming no time lag (*r*^2^ = 0.093; *p* < 0.1). By lagging active mine area by 5 years, however, the model fit dramatically improved (*r*^2^ = 0.68; *p* < 0.0001) (Fig 5); the correlations for 4 and 6 years were similar in fit, so 5 years represents the average time for the imprint of coal extraction to remain on the landscape, as defined by our NDVI thresholds. In other words, the amount of coal produced today can predict the amount of area that will be categorized as “active mining” 5 years into the future.

Using this 5-year regression model, we determined that across Appalachia for the period from 1985 through 2015, for every metric ton of coal produced, approximately 12 m^2^ of land is actively mined. This statistic does not indicate whether the 12 m^2^ land disturbance is area previously mined or not, but nonetheless indicates the active mine area necessary to produce a certain amount of coal. For comparison, a previous study of Appalachian surface mining used a decadal mine extent dataset to determine that a 1 metric ton production of coal was associated with 0.96 m^2^ of land disturbance [3]. That our model points to greater than a magnitude more of area mined per metric ton is likely explained by a difference in methods and study area. The prior study [3] regressed total mine production per county over a 20-year period against disturbed mine area, whereas we regressed yearly mine production across the entire study area against yearly disturbed area. Moreover, the prior study [3] only investigated counties in Kentucky and West Virginia, the two states with the greatest coal production and mining area, whereas we also included counties in Virginia and Tennessee. In short, we have a finer temporal resolution but a wider spatial extent, indicating typically across Appalachia that 12 m^2^ of active mine area is necessary per metric ton of coal. For the coal-rich states of Kentucky and West Virginia, however, perhaps less land area is required for the same quantity of coal.

**Fig 6:**
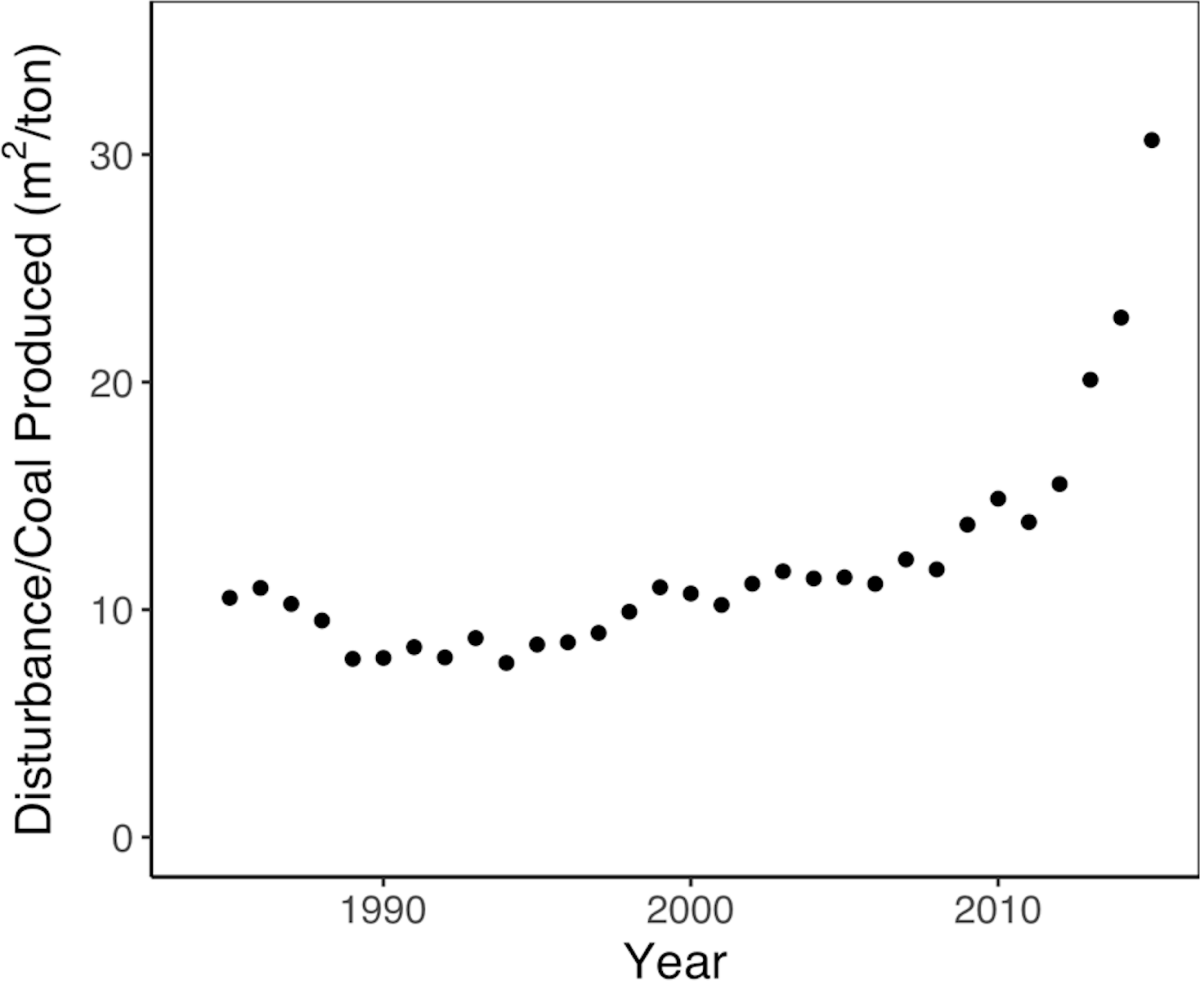
Ratio of active mine land per metric ton of coal produced over time.

We also investigated the trend of this ratio of active mine area per metric ton of coal by year (Fig 6). We find this ratio remains relatively low from 1985 through approximately 1997, meaning coal companies were extracting each metric ton of coal over relatively little land, approximately 10 m^2^. Around 1998, this ratio begins to grow quickly, suggesting coal companies had to mine more land than before to attain the same 1 metric ton of coal. By 2010, approximately 15 m^2^ of land was needed per metric ton, and by 2015, that area shot up to greater than 30 m^2^ per metric ton—or 3 times more area per metric ton than in the early 1990s.

This changing ratio over time reflects the well-documented factor of reserve depletion in Central Appalachia. Geologists have predicted since the mid-1990s that as the thickest and shallowest seams in Central Appalachia are mined out, stripping ratios (i.e., the volume of excess spoil generated per ton of coal produced) would increase, making surface mining more expensive in the region and ultimately leading to declining production as mines fail to compete with mines in other regions with lower stripping ratios [33].

The changing ratio over time could also explain why we previously found the overall 12 m^2^ of active mine necessary per metric ton of coal whereas the aforementioned prior study [3] found 0.96 m^2^. Since they only investigated the period from 1985 through 2005, they did not consider the sharp rise in the production-area ratio occurring after 2007; indeed, ratio values we find for years around 1990 are closer to the ratio value they reported. The most recent, dramatic increases may have been large enough to swing our overall ratio value up to 12 m^2^. Nevertheless, this example further demonstrates the utility of having yearly mining extent data. Even if from 1985 through 2015, ~12 m^2^ of land was needed to produce 1 metric ton of coal, our yearly mine area data shows how that average value fluctuated considerably over that time period.

### Dataset limitations

We highlight some considerations and limitations in regard to using the cumulative and annual mining datasets. First, these datasets cannot indicate the exact day or month when a given pixel started or ceased hosting mining activity because the greenest-pixel composites pull the highest NDVI value per pixel per year, even if mining started on that pixel over the course of that year. Our model calls a pixel a mine if the maximum NDVI at that pixel was consistently low over the entire year; i.e., that the pixel was an active mine during the entire year of interest.

Second, the number of images per year covering any pixel varied considerably by year, ranging from one or two images in one year to more than 30 in another; some pixels had no cloud-free images in certain years (S1 Fig). Third, our urban/water mask does not completely exclude non-mine, low-NDVI areas, due both to the data quality of the datasets comprising the mask, and due to certain landscapes (e.g., tilled fields or deforested land) also possessing low NDVI values.

And fourth, the definition of what constitutes an “active” mine is more qualitative than quantitative. In this study, we did present a quantifiable definition so that our model could run automatically (i.e., a mine is “active” if it has a low NDVI in comparison to its surroundings), but this definition does not speak to what sort of mining activity was actually taking place on that land in any given year. We merely offer our model results here to indicate approximately when and where mining activity was occurring.

## Conclusions

We demonstrate that an NDVI-based model executed through Google Earth Engine can efficiently and accurately determine yearly surface mining extent across Central Appalachia. As this is the first mining dataset that we know of possessing both a high temporal and spatial scale, and because it greatly improves upon the MTM2009 dataset, we foresee many, varied uses of the data and the processing model. To facilitate future research and to encourage scientific, computational reproducibility [34], we make the data (S1 File) and processing scripts (S2 File) freely and openly available using the Apache Version 2.0 license [35] (Table 1). These data can be updated annually as new Landsat images become available.

**Table 1:** Available processing scripts and data products for download.

Dataset or Script Name	Description
EE_Scripts/annualMiningArea.js	Converts annual greenest-pixel composites into binary active mining dataset; second part of the main processing script
EE_Scripts/annualThresholdImages.js	Determines yearly, county-scale NDVI thresholds
EE_Scripts/countInputImages.js	Identifies number of input Landsat scenes used per year in any location
EE_Scripts/exportImageryAccuracyAssessment	Exports Landsat and NAIP imagery used for accuracy assessment
EE_Scripts/greenestCompositesToAssets.js	Takes raw input Landsat imagery and creates annual greenest-pixel composites; first part of the main processing script
Mining-Cumulative	Singular dataset from 1985–2015 of all detected mining; available in raster and vector format
Mining-Yearly	Annual datasets for 1985–2015 of active mining in the given year; available in raster and vector format
Other-Data/2015_Input-Mask	Urban area, water, and road mask used in analysis
Other-Data/First-Mined	Integer raster depicting when a given pixel was first detected as a mine by our model; this dataset includes the pre-1976 and 1984 subset of the MTM2009 data
Other-Data/Last-Mined	Integer raster depicting when a given pixel was most recently detected as a mine by our model; this dataset includes the pre-1976 and 1984 subset of the MTM2009 data
Other-Data/Mine-Permits	Known surface coal mine permit boundaries clipped to the study area
Other-Data/Study-Area	Depiction of our study area; available in raster and vector format

All products may be accessed at Figshare (S1 File) or at https://www.skytruth.org/mtr-data-files/; processing scripts area also separately available (S2 File). An online, interactive visualization may be accessed at http://skytruthmtr.appspot.com/.

While we avoid hypothesizing about specific uses of the data, we do offer some suggestions about how the dataset itself could be improved and expanded upon in the future. Of greatest importance is increasing the annual dataset’s temporal scope to include 1972 through 1983, years in which the Landsat Multispectral Scanner satellites (i.e., Landsats 1, 2, and 3) were collecting data. Such a change would invalidate the need to supplement pre-1976 through 1984 with the MTM2009 data. We considered running our model on those early sensors and their respective scenes, but ultimately decided against it because these satellites’ varying spectral resolutions, lower collection frequency, and coarser pixel resolution would have necessitated the creation of a secondary model to properly locate mines. But nevertheless, these sensors do provide useful data, and Earth Engine does host their images, so future model improvements could aim to incorporate these years.

A second model extension involves looking at each Landsat image individually rather than as yearly composites. Our current model uses yearly composites to generate cloud-free images that plainly contrast high-NDVI areas (forests) with low-NDVI areas (mines). However, using yearly composites means we cannot capture exactly when a certain mine pixel began or ended its time as an active mine; our model instead labels a pixel as a mine for a given year if that pixel was an active mine during that entire year. Contrastingly, extending the model to look at each image individually means we can chart the change in each specific pixel’s NDVI values over time. Within an approximately two-week window (the collection period of Landsat images), the model could detect a sudden and consistent drop in NDVI values for a pixel, followed later by a gradually increasing NDVI. This period of sharp drop and slow rise would represent the exact time frame over which a pixel was an active mine—and could show mine recovery over time.

Finally, the model could be scaled up to find mines across the entirety of Appalachia, or perhaps over any surface coal mining region. We focused on Central Appalachia as this region has historically contained the majority of surface mining in greater Appalachia, especially mountaintop mining, but surface mining has also occurred in states including Ohio and Pennsylvania. Future model enhancements could actually quantify the spatial and temporal extent of mining across all Appalachia; the Earth Engine platform likely is able to handle this upscaling in areal extent.

In this dawning era of “big data,” regional, decadal land use change analyses that once required months of processing time on supercomputers can now be accomplished via a few lines of script and a cloud computing environment. Here we show that currently available tools and remotely sensed imagery can map surface coal mining over an 83,000 km^2^ area for 31 consecutive years. We make the data and scripts freely available so as to enable future scientific research and public advocacy. And we present a framework for future studies regarding large-scale ecological disturbances. Phenomena like urbanization, desertification, forest loss, or water quality change can all be seen via remotely sensed imagery; analyses such as the one we describe here demonstrate the relative ease of revealing the trends behind those big data.

## Supporting information

S1 File Directory of study files uploaded to Figshare. Available files on Figshare include processing scripts, input datasets, and model-produced output datasets. The directory also lists URLs and DOIs for each uploaded file.
(CSV)

S2 File Archive of all JavaScript processing scripts used in this research. These JavaScript files make heavy use of the Google Earth Engine application programming interface and thus require the freely-accessible Earth Engine platform to execute them.
(ZIP)

S3 File Detailed methods and additional discussion. Expounds upon the methods and discussion presented here.
(PDF)

S1 Table Annual accuracy assessment values. (PDF)

S1 Fig Comparison of input Landsat scene count for 1989 and 2015. These images count the number of scenes available per pixel after we had performed the cloud cleaning algorithm. The thicker white line represents the limit of our study area; the thinner white lines represent the overlapping boundaries of the Landsat scenes.
(PNG)
