# Supplementary material for: Mapping the yearly extent of surface coal mining in Central Appalachia using Landsat and Google Earth Engine
Source: PLoS One. 2018 Jul 25;13(7):e0197758. doi: 10.1371/journal.pone.0197758 (PMC6059389; doi:10.1371/journal.pone.0197758)
Supplement: S3 File — Expounds upon the methods and discussion presented here. (PDF) [file pone.0197758.s003.pdf]

Mapping the yearly extent of surface coal mining in Central Appalachia using Landsat and Google Earth Engine

Detailed methods and additional discussion

Andrew A. Pericak[1], Christian J. Thomas[2], David A. Kroodsma[2], Matthew F. Wasson[3], Matthew R. V. Ross[1], Nicholas E. Clinton[4], David J. Campagna[5], Yolandita Franklin[2], Emily S. Bernhardt[1], John F. Amos[2]

[1] Department of Biology, Duke University, Durham, North Carolina, United States of America

[2] SkyTruth, Shepherdstown, West Virginia, United States of America

[3] Appalachian Voices, Boone, North Carolina, United States of America

[4] Google Earth Engine Team, Google Inc., Mountain View, California, United States of America

[5] Department of Geology & Geography, West Virginia University, Morgantown, West Virginia, United States of America

Materials and methods

Landsat data

As the only long-term, high-resolution remote sensing platform available, we employed the full suite of Landsat data spanning from 1984 through 2016. The Landsat satellites collect multispectral data at 30 m resolution for each location on the Earth's surface every 16 days [1, 2], providing an ideal dataset for identifying land use change such as surface mines. While the United States Geological Survey (USGS) offers a variety of pre-processed Landsat data products, we used the raw, orthorectified scenes.

We created a single collection of all 10,240 Landsat scenes that intersected our study area over the period 1984 through 2016. As we were interested in annual-scale detection, we filtered data from specific Landsat satellites to the beginnings and endings of calendar years (Table 1). We note especially for scenes from Landsat 4 that we do not have scenes from every 16-day collection period simply because the satellite was not collecting data consistently or across all land area on Earth. We also point out that Google continually adds new Landsat data to the Earth Engine platform as the USGS makes those data available, meaning the two sensors still currently in operation (Landsats 7 and 8) can actually deliver nearly real-time data to our model.

Table 1. Landsat data sources.

Sensor	Spatial Resolution (m)[a]	Temporal Resolution (d)	Filtered Date Range
Landsat 4 TM[b]	30	16	January 1, 1984 - December 31, 1993
Landsat 5 TM[b]	30	16	January 1, 1984 - December 31, 2011
Landsat 7 ETM+[c]	30	16	January 1, 1999 - present
Landsat 8 OLI[d]	30	16	January 1, 2013 - present

The “Filtered Date Range” represents the scene collection dates per sensor we used for this study. We did not use Landsat 6 ETM because that satellite failed to reach orbit.
[a]These are the spatial resolutions for the visible and near-infrared bands of the respective sensors; spatial resolutions for other bands not used in this study are higher.
[b]TM = Thematic Mapper
[c]ETM+ = Enhanced Thematic Mapper Plus
[d]OLI = Operational Land Imager

Analysis model

With the collection of Landsat data, processing entailed cleaning each scene for data abnormalities or cloud cover; determining the normalized difference vegetation index (NDVI) per scene; deriving a greenest-pixel (maximum NDVI) composite per year; and labeling each pixel within each composite as likely active mine or likely non-mine based on an annual, county-scale NDVI threshold. We define active mine areas as those locations with an algorithmically-determined low NDVI as compared to the NDVI of nearby areas. Our output dataset is thus a series of annual, 30 m, binary images depicting locations where mining likely occurred throughout the given year.

While many vegetation-based indices exist, we chose to use NDVI as the basis for setting the mining threshold because of its applicability to our study goals and its simplicity in interpretation. The NDVI is a normalized, pixel-scale ratio of red light and near-infrared light, with the formula $(NIR - R) / (NIR + R)$ where “NIR” is the near-infrared band and “R” is the visible red band [3]. Since vegetation absorbs red light and reflects infrared light [3], a vegetated pixel will have a low R value but a high NIR value, yielding a high NDVI value. The standard land cover in Central Appalachia is dense, mixed forest [4]; according to the 2011 National Land Cover Database, 69% of our study area is deciduous forest, 4% is mixed forest, and 2% is evergreen forest [5]. We thus expect to see high NDVI values across the landscape, especially in relatively undisturbed areas. Conversely, a surface mine pixel will reflect most incident light in all bands,

yielding a low NDVI value. In comparison to the background forested landscape, mines are distinct against a vegetated background (Fig 1). Moreover, all Landsat sensors contain the *NIR* and *R* bands, so we could run this calculation for every scene in our collection. Since we intended to show the locations of surface mines, NDVI can efficiently and easily lead to this goal.

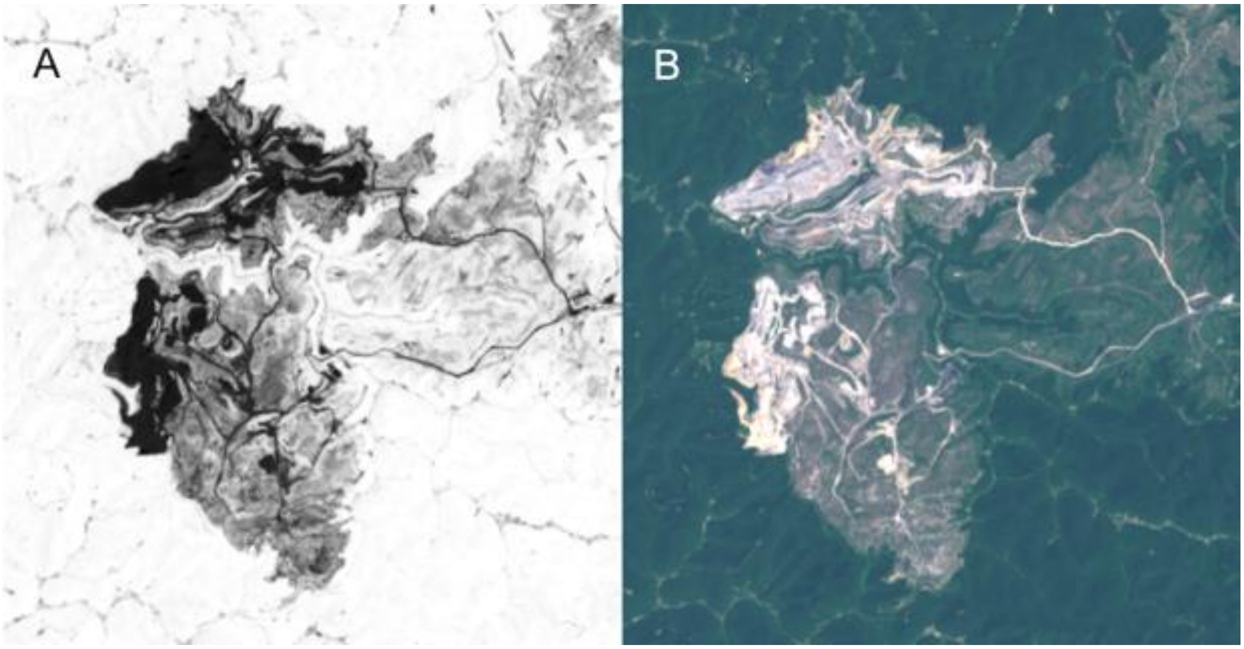

Fig 1. Example NDVI image and associated true color image.
These images from May 2014 show the area near Spurlockville, WV, and in particular the Hobet-21 mountaintop mine. Darker colors in the NDVI image (A) indicate lower NDVI values. True color imagery (B) is from Landsat 8.

Preprocessing

To each raw Landsat scene in our collection, we applied a series of preprocessing algorithms. For scenes from all sensors, we removed highly-saturated pixels, or those pixels representing the top two percent of raw values possible for that sensor. For example, the Landsat 5 TM sensor collects raw digital number values from 0 to 255, so we removed any pixels with any band values greater than 250 so as to keep the bottom 98% of the pixel value range. These high-value pixels represented data error, very bright clouds, or snow, in any case undesirable pixels for a land use analysis.

We applied Earth Engine's built-in "simpleCloudScore" algorithm to identify likely clouds. This algorithm uses pixel brightness values, temperature values, and the normalized difference snow index to give the probability that a given pixel is a cloud. We qualitatively found that assigning a threshold probability of 0.2 successfully identified most clouds while keeping non-cloud pixels.

NDVI calculation and compositing

After excluding noise and clouds, we calculated the NDVI per pixel and used those NDVI values to create annual greenest-pixel composites. We applied the NDVI formula described above to each pixel in each image, and further subset our image collection to only include images collected during the months of May through September. This filtering was designed to remove any images when trees had not yet leafed out; for instance, in some years the only cloud-free scenes over a particular region were from the winter months, meaning the background NDVI values would also be low and thus more challenging to differentiate between mine and non-mine pixels. Consequently, we obtained 4,845 cloud- and error-cleaned scenes, reduced from the 10,240 raw images in the collection.

We then created annual greenest-pixel composites by looking at all remaining scenes for a given year, finding per pixel the scene with the highest NDVI value, and compositing those pixels into one image. The resulting image could therefore contain pixels from many different dates throughout the year, but each pixel would have the greatest sampled NDVI value for that year at that location. By choosing the maximum NDVI value, mines that were established over the course of that year would likely not appear in the NDVI image; a green, forested pixel from earlier that year would have a higher NDVI value than the mine pixel, and thus the greenest-pixel composite would report the NDVI of the forested pixel. Hence, a pixel that we ultimately identify as a mine

had to have been a mine for the entirety of the given year. However, this does not mean that mining began in that pixel in that calendar year, but rather could have started in the prior year—even if it did not appear as a mine in that prior year's classification.

Urban / water mask

We aggregated public, spatial data describing urban areas, roads, and water bodies present in our study area to form a processing mask. These land covers tend to exhibit low NDVI values, since they contain little to no vegetation, so in an NDVI image they would also stand out in comparison to forest cover. To prevent our model from counting these pixels as mines, we combined various, publicly-available spatial datasets from the U.S. Census Bureau together to form a processing mask (Table 2). To account for features not exactly covered by the input data, we buffered the mask by 60 m; for instance, buffering road features delivered as lines turned those features into polygons completely overlaying the road surface. We chose a 60 m buffer distance so as to use a multiple of the 30 m Landsat scale, and because we noted qualitatively that larger multiples of 30 removed too much impervious land cover when applying the mask. By using this mask to exclude these areas from analysis, we only needed to perform a binary classification over the remaining area: land that was either mine or vegetated.

Table 2. Urban / Water Mask Data Sources.

Dataset Title[a]	Description of Data
U.S. Urban Areas	Town and city area extents
Area Hydrography	Areal water features, such as ponds and lakes
Linear Hydrography by State	Delineation of non-areal water features
All Roads by State	Delineation of state roads

[a]All data from the U.S. Census Bureau TIGER/Line Database as updated in 2015. We used 2015 as it was the most-recently updated data available.

Pixel thresholding

We used NDVI thresholds to classify each pixel in each greenest composite image as either mine or non-mine. We first assessed whether using NDVI thresholds, a computationally and conceptually simple method, could yield classification results on par with traditional, supervised classification methods. We established three, 259 km^2 (100 mi^2) study plots in Appalachian areas containing some active mining in 2005, and acquired Landsat 5 TM greenest-pixel composites for those study plots. Using the NDVI formula mentioned above, we derived NDVI per pixel for these Landsat scenes. We then randomly distributed 300 points in each plot; manual classification of the points via the Landsat scenes labeled each point as active mine or other land cover. We set a random NDVI threshold, sorting each pixel as either active mine if the NDVI of the pixel was less than the threshold, or as other land use if the pixel exceeded the threshold. Using this arbitrary classification, we then determined the overall classification accuracy of the points as if they had been classified using this NDVI threshold. We adjusted the NDVI threshold until we minimized overall error. We likewise used the "MTM2009" data—i.e., a spectral-based, supervised classification [6]—representing 2005 to classify the points, essentially assessing the accuracy of the MTM2009 dataset. We compared the accuracy results of the NDVI threshold to those of the MTM2009 data (Table 3). We found no significant difference in the two methods' ability to correctly predict active mining areas ($p > 0.51$), nor in their ability to predict non-mine areas ($p > 0.13$). These results suggest an NDVI threshold can classify mine areas with accuracies similar to intensive, supervised classifications, supporting our decision to use an NDVI threshold for the entirety of Appalachia.

Table 3. NDVI thresholding proof-of-concept accuracy results.

Accuracy Metric[a]	Manual Classification[b]	NDVI Classification	Supervised Classification

True Presence	11.22% ± 3.27%	9.33% ± 3.06%	9.33% ± 4.91%
True Absence	88.78% ± 3.27%	84.78% ± 2.71%	75.33% ± 2.40%
False Presence	N/A	4.00% ± 0.67%	13.44% ± 2.27%
False Absence	N/A	1.89% ± 1.07%	1.89% ± 1.64%

[a]Each metric in the table represents the mean value of the respective metric from the three study plots.
[b]In this column, we are merely showing what percentages of our manually-classified points fell into either the mine category (true presence) or the other land use category (true absence.)

Knowing that NDVI thresholds could deliver similar results to supervised classification, we then extended the concept to cover our entire study region. Acknowledging that we would need many different thresholds over time and space, we created annual, county-scale NDVI thresholds based off the 0th to 3rd percentile mean of presumed non-mine, forested pixels from the greenest pixel composites. Within each county and for each year, we collected NDVI values of each pixel that was not within a known mine permit boundary, and that was not within the urban/water mask, ideally leaving only forested pixels. We then subset the NDVI values only from the 0th through 3rd percentile and determined the mean of that subset's values. The resulting value became the NDVI threshold for that county and year. We used a county scale for setting this threshold to take into account the variation in local land cover and in pixel values collected per scene; because Landsat scenes in different paths are not collected on the same day, a neighboring scene might exhibit slightly different NDVI values. We opted for the mean of the 0th through 3rd percentile of forested pixels, rather than the minimum value, to account for non-forest discrepancies like mining outside of permit boundaries or urban areas not captured by the mask.

We qualitatively and quantitatively assessed other thresholding methods. We initially used one, region-wide threshold for all years, but variances in Landsat image quality and quantity yielded differing NDVI values over time and space, meaning a common threshold overestimated

mine extent in some locations and underestimated in others. We also tried a region-wide but yearly threshold, set via the Otsu method of automatic image classification [7]. This method improved results but still presented too many regions of error. We finally explored annual thresholds per county, testing various NDVI percentiles and percentile range means. We quantitatively compared areal results to independently-created mining datasets, most notably the MTM2009 dataset, and qualitatively compared these results to the MTM2009 data and to the greenest-pixel images. We determined that using the 0th to 3rd percentile range mean most closely approximated these prior datasets.

Once these thresholds were established, we labeled each pixel per year as either likely active mine or likely non-mine. A pixel with an NDVI value less than the threshold was labeled as mine, whereas a pixel with an NDVI value greater than or equal to the threshold was labeled as non-mine. In short, we most narrowly define an active mine as a location where the maximum NDVI over the course of the year was less than the NDVI threshold set in that county per that year. As we ran this classification for each greenest pixel composite image, our resulting image collection included 33 images (1984 through 2016 inclusive) describing active mining in that year.

Post-processing

The resulting binary classification images still required some cleaning to remove small pixel areas or to “fill in” gaps created by the initial cloud filtering, so we ran a few additional algorithms to improve the classification quality. We first removed any null values in a yearly classification image by looking at the images immediately prior and future. A null value in the image for year n represented a pixel over which cloud cover happened to exist for each input image. If that same pixel was labeled as a mine in the images for years (n-1) and (n+1), then we classified the pixel for image n likewise as a mine. This algorithm assumes that a mine does not

cease and become covered with vegetation over the course of one year. In order to remove any null pixels remaining after running this algorithm, we labeled all remaining null pixels as non-mine, which in some cases might lead to underestimation of the actual mine extent for that year.

We performed a similar process to remove noisy pixels in the classification images. If a pixel was labeled as a mine for year *n* but was labeled as non-mine for years (n-1) and (n+1), then we relabeled the pixel at year *n* as non-mine. And conversely, if a pixel was labeled as non-mine at year *n* but mine in years (n-1) and (n+1), then we relabeled the pixel for year *n* as mine. For both this noise cleaning method and the null removal method, we could not run the algorithms on the greenest-pixel composites for 1984 and 2016 since we had, respectively, no prior or future image.

Finally, we removed any pixels falling within the urban/water mask and removed any mine pixel clusters with fewer than 10 pixels (patches less than 9,000 m^2). The former operation ensured that the model, in finding low NDVI pixels, only captured likely mine pixels. The latter operation removed most remaining sources of noise, while acknowledging that some forms of surface mining (such as contour) may have been detectable only in small patches.

Years analyzed

While we intended to present results from our model for each year from 1984 through 2016 inclusive, we ultimately present in the primary text results only from 1985 through 2015 inclusive, or 31 consecutive years. We omitted 1984 and 2016 from our results because we could not apply the post-processing cleaning methods to these years, as described above. Running the model on these years yielded mine extent values much higher than those in neighboring years; while such high values could represent real conditions, the fact that these years were not cleaned means we cannot confirm or deny those results. In order to ensure the greatest model accuracy, we decided to omit those years. While 1984 will therefore always remain omitted from the dataset, 2016 can

be added once images from May through September 2017 have been collected, because 2016's "future" imagery will then exist.

Accuracy assessment

Given the large spatial and temporal scope of our model, we assessed the classification accuracy annually across the entire study area. We established 10, 250 km^2, circular plots randomly located throughout the study area, ensuring that each study plot contained some active mining throughout the 31-year period of 1985 through 2015 (Fig 2**)**. A set of 5,000 randomly distributed points was created for each study plot. We acquired annual visible-color (RGB) composite imagery from the National Agriculture Imagery Program (NAIP), when available, and otherwise yearly RGB composites from Landsat data. For each plot and per year, we manually classified a minimum of 2,000 points as either mine or non-mine based on visual interpretation of the RGB composites, totaling at least 620,000 classified points. We randomly sampled each plot's 2,000 points to create a subset consisting of 150 non-mine points and 50 mine; where necessary, we classified additional random points to attain 50 mine points per plot per year. This subset of points, totaling 62,000 classified locations over 31 years, served as our final accuracy assessment dataset. From this final dataset, we assessed the model accuracy on an annual basis, only using the classified points for that given year.

Figure 2. Accuracy Assessment Study Plots

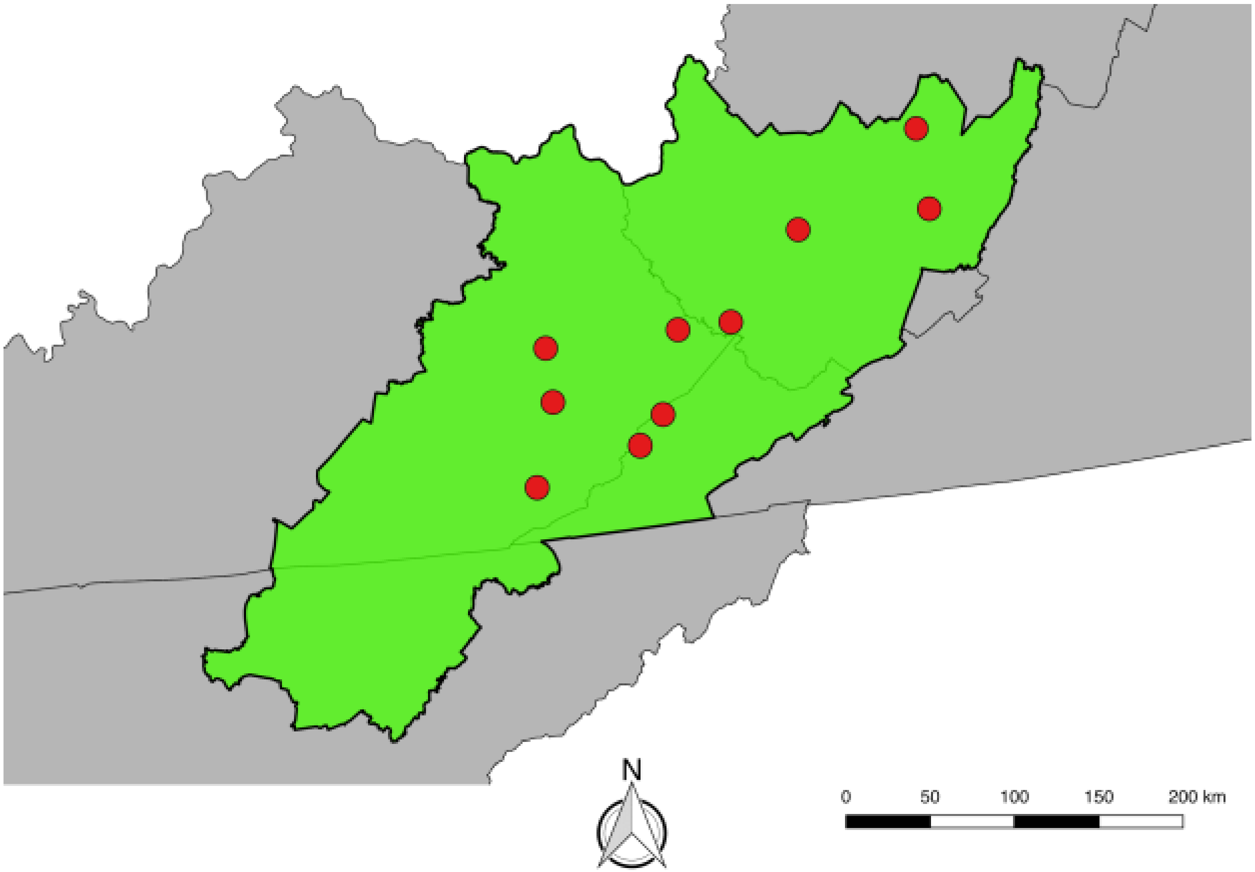

Incorporating MTM2009 data

Since our model started at 1985 but we know mountaintop surface mining with valley fills has occurred prior to that date, we incorporated the 1976 through 1984 subset of the MTM2009 data into our dataset. We selected only those pixels identified as mines in 1976 or 1984 from the MTM2009 data and spatially appended them to our 1985 through 2015 cumulative mining dataset. We thus generated a "first-mined" dataset that reveals whether a certain area was first converted into a surface mine either by 1976, between 1977 and 1984, or in any year from 1985 through 2015. Of note, many mines labeled by MTM2009 as "1976" likely started at some unknown date prior to 1976, so we cannot precisely say when those earliest mines began. We likewise generated a "last-mined" dataset that says when a given area was most recently an active mine; however, this

dataset cannot show if a given area was once mined but became reclaimed (i.e., ceased mining) and later became a mine again. Our results in the primary text are based off either the “first-mined” dataset or the annual mining dataset generated in this study alone.

Results and Discussion

Accuracy assessment results

We find that our NDVI-based model accurately and efficiently reveals yearly mining extent (Table 4). We first note the values for Cohen’s Kappa coefficient, which range from 0.62 to 0.93 per year. These positive values suggest that, in all cases, our model performs 62 to 93 percent better than random chance. That some years have relatively lower values for Kappa than others likely reflects discrepancies in image quality among years; the lowest Kappa values tend to correspond to years where fewer cloud-free images existed during summer months, meaning the derived greenest-pixel composites may have missing values. While annual overall accuracy values are high, we note that these values are likely skewed because our manually classified dataset has three times as many non-mine points as it does mine points. Of greatest importance for the goals of this study, therefore, is the user’s accuracy of mined points; this metric reveals how accurate the resulting classification is at finding mines in particular. For each year, this value is at least 0.83 or higher, indicating that at least 83 percent of pixels labeled as mines actually represent mines on the ground. Of the 31 years analyzed, 21 years have mine user accuracy values greater than or equal to 0.9. As before, the years with the lowest values tend to be the years with lower-quality greenest-pixel composites.

Table 4. Annual accuracy assessment values.

Year	Overall	Cohen’s	Producer’s -	User’s -	Producer’s -	User’s -

	accuracy	Kappa	non-mine	non-mine	mine	mine
1985	0.90	0.68	0.98	0.89	0.62	0.92
1986	0.92	0.76	0.98	0.92	0.72	0.93
1987	0.93	0.78	0.99	0.92	0.73	0.95
1988	0.92	0.76	0.98	0.92	0.73	0.92
1989	0.89	0.67	0.98	0.89	0.63	0.90
1990	0.93	0.78	0.99	0.92	0.74	0.95
1991	0.91	0.74	0.98	0.92	0.71	0.90
1992	0.93	0.78	0.97	0.94	0.77	0.90
1993	0.94	0.83	0.98	0.94	0.80	0.94
1994	0.91	0.73	0.98	0.91	0.68	0.92
1995	0.92	0.77	0.98	0.92	0.74	0.92
1996	0.90	0.71	0.97	0.91	0.69	0.88
1997	0.91	0.75	0.97	0.92	0.73	0.91
1998	0.93	0.81	0.96	0.95	0.84	0.86
1999	0.91	0.75	0.96	0.93	0.76	0.85
2000	0.94	0.83	0.96	0.96	0.87	0.86
2001	0.92	0.78	0.97	0.93	0.78	0.89
2002	0.88	0.62	0.98	0.87	0.56	0.91
2003	0.89	0.66	0.98	0.88	0.61	0.91
2004	0.90	0.71	0.96	0.91	0.70	0.86
2005	0.92	0.79	0.95	0.94	0.83	0.86
2006	0.93	0.81	0.94	0.96	0.89	0.83
2007	0.93	0.81	0.94	0.96	0.89	0.83
2008	0.93	0.82	0.95	0.96	0.88	0.86
2009	0.92	0.76	0.98	0.91	0.72	0.94

2010	0.92	0.78	0.95	0.95	0.82	0.85
2011	0.94	0.84	0.98	0.95	0.84	0.92
2012	0.93	0.80	0.98	0.93	0.77	0.94
2013	0.92	0.79	0.97	0.93	0.79	0.89
2014	0.94	0.84	0.98	0.95	0.84	0.92
2015	0.93	0.79	0.98	0.93	0.76	0.92

Dataset limitations

We highlight some considerations and limitations in regard to using the cumulative and annual mining datasets. First, as previously mentioned, the dataset cannot indicate the exact day or month when a given pixel started or ceased hosting mining activity. The greenest-pixel composites, which serve as the basis for the model's classification, pull the highest NDVI value per pixel per year, regardless of what day-of-year the Landsat sensor collected that highest NDVI pixel. For instance, if mining started in a pixel in August (and thus the pixel has a low NDVI value at that time), the greenest pixel composite would most likely not reveal the start of this mining in that year since greater NDVI values (i.e., a forested landscape) had probably already been measured earlier that year. In short, our model calls a pixel a mine if the maximum NDVI at that pixel was consistently low over the entire year; i.e., that the pixel was an active mine during the entire year of interest.

Second, as previously mentioned, some years had few images with which to make a greenest pixel composite, or had images with non-removable errors. For instance, in 1989 many locations in our study area were only described by one or two cloud-free images, meaning the greenest pixel composite only had those images' pixel values to choose from (Fig 3). Notably, an advantage to using an annual composite image is that at minimum one "good" image need exist

per pixel so as to have a composite spanning the study area. Nevertheless, some of 1989’s pixels have no coverage whatsoever, so the model can only say if those pixels were mines if they were likewise mines in the years previous and future (via the null pixel cleaning algorithm).

Fig 3. Comparison of input Landsat scene count for 1989 and 2015.

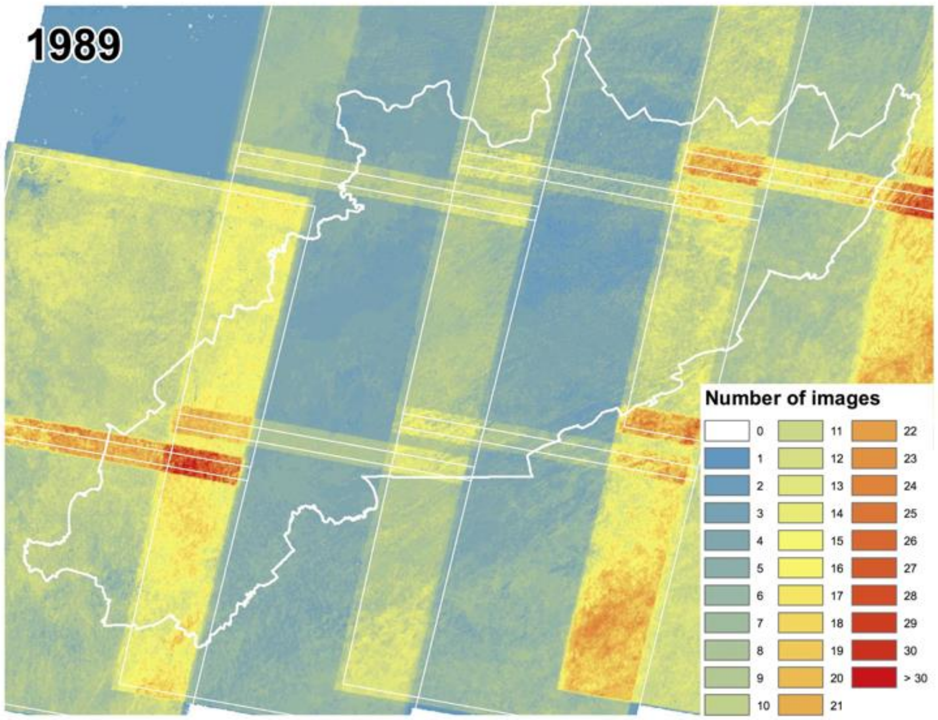

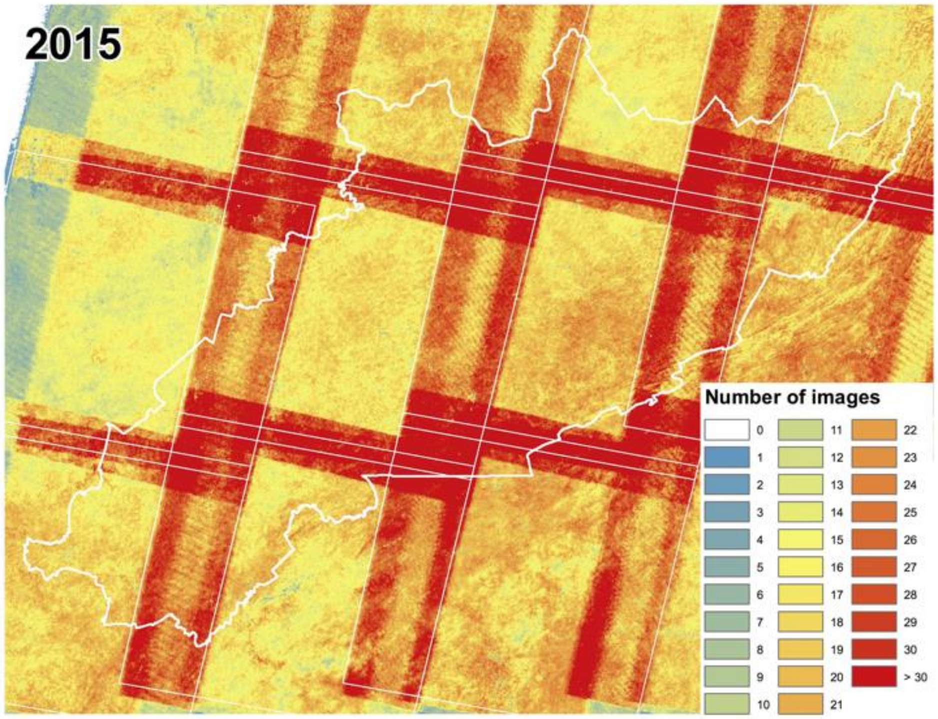

These images count the number of scenes available per pixel after we had performed the cloud cleaning algorithm. The thicker white line represents the limit of our study area; the thinner white lines represent the overlapping boundaries of the Landsat scenes.

Third, our urban/water mask does not completely exclude non-mine, low-NDVI areas. The mask is only as good as the background data we used to make the mask in the first place, and so any omissions in those background datasets will likely show up as mines in our model output. For instance, tracts of land that were deforested over some year, perhaps to harvest timber, occasionally show up as mines because of their low NDVI values; similarly, barren agricultural land near the edges of our study area also sometimes appear as mines. No systematic, automated method exists to remove these sporadic errors and varying land classes, so in cases where they occur they remain in the output dataset.

Finally, the definition of what constitutes an “active” mine is more qualitative than quantitative. In this study, we did present a quantifiable definition so that our model could run automatically; in our case, a mine is any pixel with a lower NDVI value than the surrounding forested landscape, or essentially any pixel without vegetation. But mine reclamation efforts may also have much mining-related activity, even if those pixels have higher NDVI values due to new vegetation. Similarly, a once-mined pixel may not yet have reclamation, but mining crews are not actively extracting coal there either. In neither example is it clear that the pixel is or is not actively being mined. We merely offer our model results here to indicate approximately when and where mining activity was occurring based on the absence of significant vegetation, i.e., low NDVI values. And we can confidently show where exists the approximate 5,900 km^2 of Central Appalachia that has ever been mined since ~1976, even if we cannot as confidently say when that mining started or ended.

References

1. Chander G, Markham B, Helder D. Summary of current radiometric calibration coefficients for Landsat MSS, TM, ETM+, and EO-1 ALI sensors. Remote Sens Environ. 2009 May; 113(5):893-903.
2. Roy D, Wulder M, Loveland T, Woodcock C, Allen R, Anderson M, et al. Landsat-8: Science and product vision for terrestrial global change research. Remote Sens Environ. 2014 April; 145:154-172.
3. Myneni R, Hall F, Sellers P, Marshak A. The Interpretation of Spectral Vegetation Indexes. IEEE Trans Geosci Remote Sens. 1995; 33(2):481-486.
4. Sayler K. Land cover trends project: Central Appalachians [Internet]. Available from: http://landcovertrends.usgs.gov/east/eco69Report.html
5. Homer C, Dewitz J, Yang L, Jin S, Danielson P, Xian G, et al. Completion of the 2011 National Land Cover Database for the conterminous United States - representing a decade of land cover change information. Photogramm Eng Remote Sensing. 2015 May; 81(5):345-354.
6. SkyTruth. Mountaintop Removal Mining, Part 1: Measuring the extent of mountaintop removal in Appalachia [Internet]. 2009 [cited 2016 Nov 15]. Available from: http://skytruth.org/2009/12/measuring-mountaintop-removal-mining-in/
7. Otsu N. A Threshold Selection Method from Gray-Level Histograms. IEEE Trans Syst Man Cybern. 1979 Jan; 9(1):62-66.
