# Supplementary material for: Mapping the yearly extent of surface coal mining in Central Appalachia using Landsat and Google Earth Engine
Source: PLoS One. 2018 Jul 25;13(7):e0197758. doi: 10.1371/journal.pone.0197758 (PMC6059389; doi:10.1371/journal.pone.0197758)
Supplement: S1 Table — (PDF) [file pone.0197758.s004.pdf]

Table S1. Annual accuracy assessment values.

Year	Overall accuracy	Cohen's Kappa	Producer's - non-mine	User's - non-mine	Producer's - mine	User's - mine
1985	0.90	0.68	0.98	0.89	0.62	0.92
1986	0.92	0.76	0.98	0.92	0.72	0.93
1987	0.93	0.78	0.99	0.92	0.73	0.95
1988	0.92	0.76	0.98	0.92	0.73	0.92
1989	0.89	0.67	0.98	0.89	0.63	0.90
1990	0.93	0.78	0.99	0.92	0.74	0.95
1991	0.91	0.74	0.98	0.92	0.71	0.90
1992	0.93	0.78	0.97	0.94	0.77	0.90
1993	0.94	0.83	0.98	0.94	0.80	0.94
1994	0.91	0.73	0.98	0.91	0.68	0.92
1995	0.92	0.77	0.98	0.92	0.74	0.92
1996	0.90	0.71	0.97	0.91	0.69	0.88
1997	0.91	0.75	0.97	0.92	0.73	0.91
1998	0.93	0.81	0.96	0.95	0.84	0.86
1999	0.91	0.75	0.96	0.93	0.76	0.85
2000	0.94	0.83	0.96	0.96	0.87	0.86
2001	0.92	0.78	0.97	0.93	0.78	0.89
2002	0.88	0.62	0.98	0.87	0.56	0.91
2003	0.89	0.66	0.98	0.88	0.61	0.91
2004	0.90	0.71	0.96	0.91	0.70	0.86
2005	0.92	0.79	0.95	0.94	0.83	0.86
2006	0.93	0.81	0.94	0.96	0.89	0.83
2007	0.93	0.81	0.94	0.96	0.89	0.83

2008	0.93	0.82	0.95	0.96	0.88	0.86
2009	0.92	0.76	0.98	0.91	0.72	0.94
2010	0.92	0.78	0.95	0.95	0.82	0.85
2011	0.94	0.84	0.98	0.95	0.84	0.92
2012	0.93	0.80	0.98	0.93	0.77	0.94
2013	0.92	0.79	0.97	0.93	0.79	0.89
2014	0.94	0.84	0.98	0.95	0.84	0.92
2015	0.93	0.79	0.98	0.93	0.76	0.92
