# Supplementary figures and images for: Mapping the yearly extent of surface coal mining in Central Appalachia using Landsat and Google Earth Engine
Source: PLoS One. 2018 Jul 25;13(7):e0197758. doi: 10.1371/journal.pone.0197758 (PMC6059389; doi:10.1371/journal.pone.0197758)

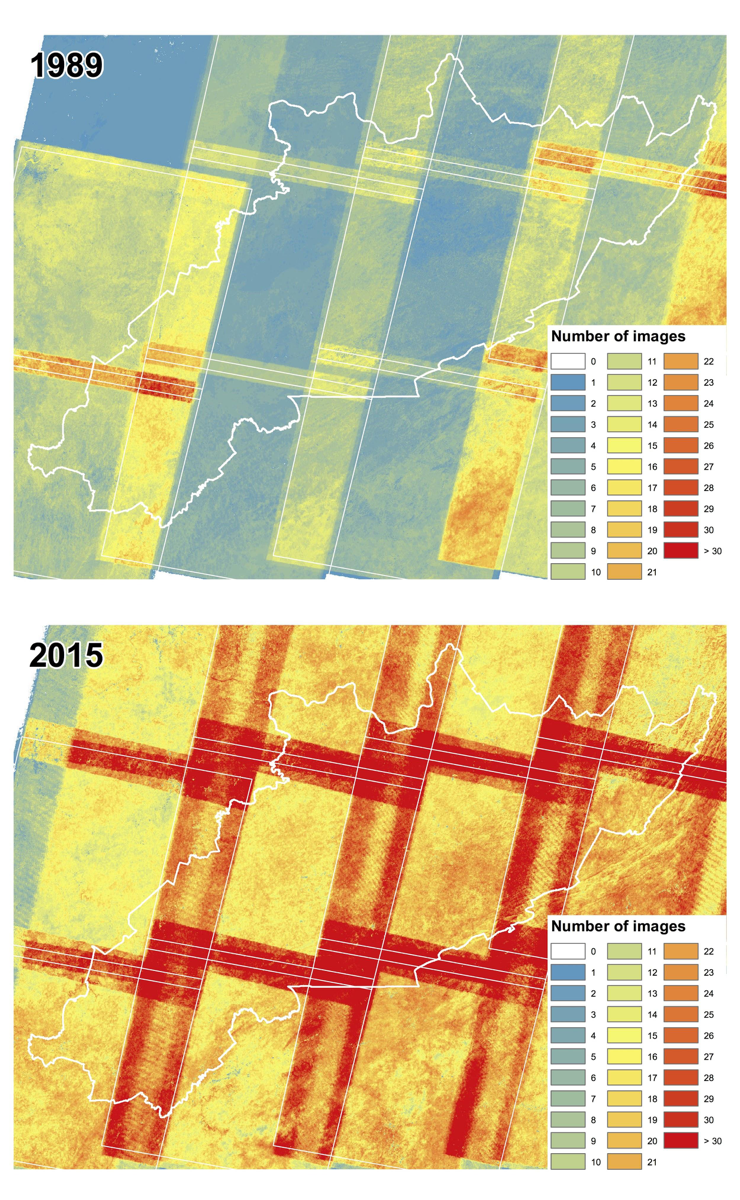

Supplement: S1 Fig — These images count the number of scenes available per pixel after we had performed the cloud cleaning algorithm. The thicker white line represents the limit of our study area; the thinner white lines represent the overlapping boundaries of the Landsat scenes. (PNG) [file pone.0197758.s005.png]
